# Geometric learning of functional brain network on the correlation manifold

**DOI:** 10.1038/s41598-022-21376-0

**Published:** 2022-10-22

**Authors:** Kisung You, Hae-Jeong Park

**Affiliations:** 1 Department of Internal Medicine, Yale University School of Medicine, New Haven, CT USA; 2 Center for Systems and Translational Brain Science, Institute of Human Complexity and Systems Science, Yonsei University, Seoul, Republic of Korea; 3 Graduate School of Medical Science, Brain Korea 21 Project, Department of Nuclear Medicine, Psychiatry, Yonsei University College of Medicine, Seoul, Republic of Korea; 4 Department of Cognitive Science, Yonsei University, Seoul, Republic of Korea

**Keywords:** Network models, Systems analysis, Applied mathematics, Functional magnetic resonance imaging, Software

## Abstract

The correlation matrix is a typical representation of node interactions in functional brain network analysis. The analysis of the correlation matrix to characterize brain networks observed in several neuroimaging modalities has been conducted predominantly in the Euclidean space by assuming that pairwise interactions are mutually independent. One way to take account of all interactions in the network as a whole is to analyze the correlation matrix under some geometric structure. Recent studies have focused on the space of correlation matrices as a strict subset of symmetric positive definite (SPD) matrices, which form a unique mathematical structure known as the Riemannian manifold. However, mathematical operations of the correlation matrix under the SPD geometry may not necessarily be coherent (i.e., the structure of the correlation matrix may not be preserved), necessitating a post-hoc normalization. The contribution of the current paper is twofold: (1) to devise a set of inferential methods on the correlation manifold and (2) to demonstrate its applicability in functional network analysis. We present several algorithms on the correlation manifold, including measures of central tendency, cluster analysis, hypothesis testing, and low-dimensional embedding. Simulation and real data analysis support the application of the proposed framework for brain network analysis.

## Introduction

One of the widely accepted perspectives on the human brain claims that the brain is a network consisting of interactions among distributed regions^1^. Such interactions are often captured by the correlation matrix of spontaneous fluctuations, observed in the resting-state functional magnetic resonance imaging (rs-fMRI)^2^ or electroencephalogram (EEG) / magnetoencephalogram (MEG)^3,4^. Representations of functional brain network as a correlation matrix have been central parts of analysis in diverse fields of brain research such as exploration of brain diseases^5–7^, individual identification^8^, between-individual variability^9^, and dynamic connectivity analysis^10–14^ to name a few. Studying with representation matrices are typically done by considering interactions (i.e., edges of the network) independently^15,16^ or together to explain certain dependent edges^17,18^.

One limitation of the aforementioned approaches is that it does not take an intrinsic dependence structure among edges into consideration. Mathematically, the correlation matrix is a subtype of symmetric and positive-definite (SPD) matrices, whose collection forms a unique geometric structure called Riemannian manifolds. In neuroimaging communities, SPD approach has gained popularity with successful applications such as exploratory analysis of group-level functional connectivity^19,20^, statistical hypothesis testing^21^, regression for functional-structural prediction^22^, individual fingerprinting^23^, and so on.

The geometric approach of SPD matrices has also been applied to studies with correlation matrices^19^. However, the naive SPD-based approach incurs a practical issue that cannot be easily neglected. Suppose we are given two correlation matrices $C_1$ and $C_2$. Under a variety of geometric characterizations of SPD space, a mean of two matrices may not be a correlation matrix, either numerically or analytically, except for the Euclidean geometry. Since the space of correlation matrices is a strict subset of the SPD manifold, the unsophisticated application of SPD geometry to correlation matrices does not guarantee any valid operations on the manifold to result in a correlation matrix. Therefore, it is natural to consider a more strict geometric structure regarding studies with a set of correlation matrices.

Compared to the SPD manifold, the space of correlation matrices known as elliptope^24^ has little been studied except for a few notable works that lack desirable properties or availability of computational routines^25,26^. In response, a recent study^27^ proposed to use a quotient geometry of SPD manifold induced by affine-invariant Riemannian metric (AIRM)^28^ for the space of correlation matrices. In a layperson’s language, the induced manifold structure of correlation space inherits characteristics of the ambient SPD manifold to allow similar machinery for inference on the correlation manifold. Also, core numerical routines on the correlation manifold were elaborated under the quotient geometry^29^.

In this work, we incorporate the recent development of the quotient geometry of the correlation manifold into well-known algorithms in machine learning and statistical inference for functional connectivity analysis. The proposed algorithms over the correlation manifold include central tendency measures, cluster analysis, hypothesis testing, and low-dimensional embedding. We then demonstrate the efficacy of the proposed framework using simulated and real data examples. All the algorithms are implemented as a MATLAB toolbox (Mathworks, inc. USA) for public use, not only for functional network analysis but also for any application fields using the correlation matrix on the proper geometry.

## Methods

### Quotient geometry of the correlation manifold

Our main interest is to study the space of correlation matrices, which are symmetric, positive-definite, and have unit diagonal elements. We denote a space of $(n\times n)$ correlation matrices as ${\mathscr {C}}_{++}^n= \lbrace X \in \mathbb {R}^{n\times n}~\vert ~ X=X^\top ,~ \text {rank} (X) = n,~\text {diag}(X) = 1\rbrace$, which is of dimension $n(n-1)/2$. From the definition, we see that the space of correlation matrices is a strict subset of the SPD manifold denoted as ${\mathscr {S}}_{++}^n$. David^27^ employed a well-known result from Lie group theory^30^ to equip ${\mathscr {C}}_{++}^n$ with manifold structure. Theorem 2.1 of^27^ showed that the Lie group ${\mathscr {D}}_{++}^n$ of strictly positive entries acts smoothly, properly, and freely on ${\mathscr {S}}_{++}^n$ so that the quotient manifold ${\mathscr {S}}_{++}^n/{\mathscr {D}}_{++}^n$ is a smooth manifold that is uniquely expressed by correlation matrices. Therefore, any SPD matrix $\Sigma \in {\mathscr {S}}_{++}^n$ can be mapped to the correlation manifold by an invariant submersion $\text {diag}(\Sigma )^{-1/2} \cdot \Sigma \cdot \text {diag}(\Sigma )^{-1/2} \in {\mathscr {C}}_{++}^n\cong {\mathscr {S}}_{++}^n/{\mathscr {D}}_{++}^n$. Furthermore, a metric on ${\mathscr {C}}_{++}^n$ is induced by any metric on the ambient space ${\mathscr {S}}_{++}^n$ when invariant under the group action of ${\mathscr {D}}_{++}^n$. This enables the geometry of ${\mathscr {C}}_{++}^n$ to inherit characteristics of an ambient SPD manifold under the affine-invariant Riemannian metric in many aspects. We refer interested readers to Supplementary Information for more details on elements of Riemannian geometry and construction of geometric structure along with core computational routines on the correlation manifold.

### Measures of central tendency

We now introduce several algorithms for learning and inference a given set of correlation matrices. For the rest of this paper, we use the following notations; $\lbrace C_i\rbrace _{i=1}^N \subset {\mathscr {C}}_{++}^n$ for *N* observations of $(n\times n)$ correlation matrices, $\mu$ and $\mu _{j}$ for means of all data and class *j*, $S_i$ an index set of observations that belong to the cluster *i*, superscript in parenthesis $^{(t)}$ for indexing an iteration *t*, and $d:{\mathscr {C}}_{++}^n\times {\mathscr {C}}_{++}^n\rightarrow \mathbb {R}_+$ a distance function.

One of the primary characterizations for an empirical distribution of the data is measures of central tendency such as mean, median, and mode. For manifold-valued data, however, such entities are not trivially obtained due to the nonlinear nature of a Riemannian manifold. The Fréchet mean, also known as Riemannian center of mass, is a generalization of the aforementioned concepts to arbitrary metric spaces^31^. More generally, the sample version of the Riemannian $L_p$ center of mass $\mu _p$ is defined as a minimizer of the functional $f(\mu )$,

$$\begin{aligned} \mu _p = \underset{\mu \in {\mathscr {C}}_{++}^n}{{{\,\mathrm{argmin}\,}}}~ f (\mu )\text { where } f(\mu ) = \frac{1}{N} \sum _{i=1}^N d^p (\mu , C_i), \end{aligned}$$

for $1\le p < \infty$^32^. Given a minimizer $\hat{\mu }_p$, the sample variation $V_p = \sum _{i=1}^N d^p (\hat{\mu }_p, C_i)/N$ quantifies dispersion of the distribution. For example, when $p=2$ and data lies on $\mathbb {R}$, this corresponds to the sample variance.

For the special cases of $p=1$ and $p=2$, the minimizers are also known as Fréchet median and Fréchet mean, respectively^33^. For the Fréchet mean computation, one of the standard algorithms is Riemannian gradient descent^28^ which we adopt in our implementation (**corr_mean.m**). At the *t*-step, gradient of the cost function $f(\mu )$ is evaluated on the tangent space of $\mu ^{(t)}$ and projected back onto ${\mathscr {C}}_{++}^n$ via exponential map. To summarize, with an initial point $\mu ^{(0)}$, we repeat the following steps to obtain the sample Fréchet mean

$$\begin{aligned} \nabla f^{(t)}&= \frac{2}{N} \sum _{i=1}^N log_{\mu ^{(t)}} (C_i), \\ \mu ^{(t+1)}&= exp_{\mu ^{(t)}} (-\nabla f^{(t)}), \end{aligned}$$

until convergence. A convenient choice of stopping criterion is to iterate until $\Vert \mu ^{(t+1)} - \mu ^{(t)} \Vert _F < \varepsilon$ for a specified tolerance level $\varepsilon$. This criterion stops the algorithm by a small increment in Frobenius norm rather than the geodesic distance. We recommend this criterion for two reasons. First, computing the incremental change in Frobenius norm is much cheaper than the geodesic distance. Second, a small increment implies that the evaluated gradient is of very small magnitude so that an iterate is sufficiently close to a critical point of the functional.

The function **corr_median.m** computes sample Fréchet median using a Riemannian adaptation of the Weiszfeld algorithm^34–37^. The cost function for minimization is as follows

$$\begin{aligned} g(\mu ) = \frac{1}{N} \sum _{i=1}^ d(\mu , C_i)\quad \text {for some }\mu \in {\mathscr {C}}_{++}^n. \end{aligned}$$

Given an initial point $\mu ^{(0)}$, the Riemannian Weiszfeld algorithm iterates the following steps

$$\begin{aligned} W^{(t)}&= \frac{\sum _{i=1}^N w_i^{(t)} log_{\mu ^{(t)}} (C_i)}{\sum _{i=1}^N w_i^{(t)}}, \\ \mu ^{(t+1)}&= exp_{\mu ^{(t)}} (W^{(t)}), \end{aligned}$$

for weights $w_i^{(t)} = 1/d(\mu ^{(t)}, C_i),~i=1,\ldots ,N$. It is worthy to mention a case where an iterate $\mu ^{(t)}$ coincides with one of the observations $C_i$’s, which incurs the singularity of the corresponding weight. Common strategies to avoid such issues may include stopping an algorithm at convergence to one of the data points, adjusting a weight by adding a sufficiently small number, or removing the coinciding observations’ contributions.

### Cluster analysis

Next, we implement three clustering algorithms that partition the data into *K* disjoint subsets called clusters and two measures of cluster validity that help determine the optimal number of clusters *K* in a data-driven manner.

The *k*-means algorithm^38^ is one of the primary methods for cluster analysis, which can be easily extended to Riemannian manifolds where a routine for computing the mean is readily available. We implemented a standard version of Lloyd’s algorithm^39^ that makes an iterative refinement of the cluster assignment (**corr_kmeans.m**).

1. Select *K* data points as cluster centroids $\lbrace \mu _i^{(0)} \rbrace _{i=1}^K$.
2. Repeat the following steps until convergence:
  Compute distances from an observation to cluster centroids and make an assignment to the cluster with minimal distance. The cluster membership $S^{(t)} = [S_1^{(t)},\ldots , S_K^{(t)}]$ is given by
    $$\begin{aligned} S_k^{(t)} = \lbrace i~\vert ~ d(C_i, \mu _k^{(t)}) \le d(C_i, \mu _j^{(t)}) \text { for all }j\ne k \rbrace . \end{aligned}$$
    When an observation belongs to multiple clusters, assign it randomly.
  For each cluster *k*, update cluster centroid by Fréchet mean of corresponding observations,
    $$\begin{aligned} \mu _k^{(t+1)} = \underset{\mu \in {\mathscr {C}}_{++}^n}{{{\,\mathrm{argmin}\,}}} \sum _{i \in S_k^{(t)}} d^2 (\mu , C_i). \end{aligned}$$

The *k*-medoids algorithm^40^ is another popular partitioning method. Compared to the *k*-means algorithm, *k*-medoids use a central observation of a given cluster as a centroid that minimizes the average dissimilarities. This characteristic enables the algorithm to be used on an arbitrary metric space, not to mention Riemannian manifolds. Replacing the mean with one of the observations gives two benefits. First, the *k*-medoids algorithm is regarded to be more robust to outliers^41^. More importantly, the algorithm does not require explicit computation of cluster means as it selects observations that play the role of central objects. This is especially appealing in our context since the computation of Fréchet means on the correlation manifold involves nested iterations. Our implementation (**corr_kmedoids.m**) employs that of the original partitioning around methods algorithm (PAM)^40^, which is similar to Lloyd’s algorithm except that the update procedure for cluster centroids is performed by selecting the observations with minimal average dissimilarities.

The last clustering method we included is the spectral clustering algorithm^42–44^. Inspired by spectral graph theory^45^, spectral clustering first finds a low-dimensional embedding via the spectral information of a data-affinity graph and its Laplacian. Our implementation of the algorithm (**corr_specc.m**) makes use of data-adaptive construction for the affinity graph^46^ and the normalized symmetric Laplacian matrix^44^. The algorithm is described as follows.

1. Construct an $(N\times N)$ affinity/similarity matrix *S*,
  $$\begin{aligned} S_{i,j} = \exp \left( - \frac{d(C_i,C_j)^2}{\sigma _i \cdot \sigma _j}\right) , \end{aligned}$$
  where $\sigma _i$ is the distance from $C_i$ to its *k*-th nearest neighbor.
2. The graph Laplacian is defined as
  $$\begin{aligned} L = D^{-1/2} S D^{-1/2}, \end{aligned}$$
  where *D* is called a degree matrix with entries $D_{ii} = \sum _{j} S_{ij}$ and $D_{ij} = 0$ for $i\ne j$.
3. Denote $V \in \mathbb {R}^{N\times K}$ whose columns are *K* eigenvectors of *L* that correspond to *K* smallest eigenvalues.
4. Normalize each row of *V* by $V_{i:} \leftarrow {V_{i:}}/{\Vert V_{i:} \Vert }$.
5. Cluster assignment is obtained by applying *k*-means clustering algorithm onto the rows of *V*.

When little prior knowledge or strong assumptions are available for the intrinsic clustering structure of the data, cluster validity indices offer a way to quantify how coherent the attained clustering assignment is regarding the data^47^. Among many quality indices, we offer two of the celebrated indices, silhouette score (**corr_silhouette.m**) and Calinski-Harabasz (CH) index (**corr_CH.m**).

First, silhouette score^48^ measures proximity of observations within a cluster to its neighboring clusters. For each observation $C_i$, a silhouette value is defined as $s(i) = \{b(i)-a(i)\}/{\max \lbrace a(i), b(i) \rbrace }$ for two auxiliary quantities

$$\begin{aligned} a(i) = \frac{1}{|S_i| - 1} \sum _{j \in S_i\backslash \lbrace i \rbrace } d(C_i, C_j)\quad \text {and}\quad b(i) = \underset{i\ne j}{\min } \frac{1}{|S_i |} \sum _{j \in S_j} d(C_i, C_j), \end{aligned}$$

where $S_i$ denotes the indices that share the same label as an *i*-th observation. The quantity *a*(*i*)) measures cohesiveness of a cluster by averaging distances between $C_i$ and the rest within same cluster, while *b*(*i*) quantifies minimal degree of separation from an observation to all points in other clusters. The global silhouette score $S^* = \sum _{i=1}^N s(i) / N$ is defined as an arithmetic mean of pointwise silhouette values lying in $[-1,1]$. A partition is considered to be optimal for large values of the global silhoutte score.

CH index^49^ is represented as a ratio of degrees of separation and cohesion. For a partition of *K* disjoint clusters, denote $\lbrace \mu _k \rbrace _{k=1}^K$ and $\mu$ as Fréchet means per class and the entire dataset, respectively. The index is defined as follows,

$$\begin{aligned} CH^* = \frac{\text { degree of separation}}{\text {degree of cohesion}} = \frac{ \sum _{k=1}^K |S_k| \cdot d(\mu _k, \mu )^2 / (K-1) }{ \sum _{k=1}^K \sum _{i \in S_k} d(C_i, \mu _k)^2 / (N-K) }, \end{aligned}$$

where the terms resemble those from discriminant analysis^50^. As the definition of the index is a ratio, it is reasonable to consider a partition of higher CH index as a superior candidate for clustering as it indicates that the given partition describes the clustering structure of the data.

### Dimension reduction for visualization

In order to visualize how the set of correlation matrices is distributed, we implemented three methods for dimension reduction; classical and metric multidimensional scaling (MDS)^51^ and principal geodesic analysis (PGA)^52^.

MDS refers to a class of algorithms for low-dimensional data embedding based on pairwise similarities. Among many variants, we present classical (**corr_cmds.m**) and metric (**corr_mmds.m**) variants of MDS. We denote $X_i \in \mathbb {R}^p, i=1,\ldots ,N$ a set of low-dimensional embeddings in some Euclidean space and $D_{N\times N}$ a matrix of pairwise distances, i.e., $D_{ij} = d(C_i,C_j)$.

Classical MDS minimizes the cost function known as *strain*

$$\begin{aligned} Strain(X_{1:N}) = \sqrt{\frac{\sum _{ij} (B_{ij} - X_i^\top X_j)^2}{\sum _{ij} B_{ij}^2}}, \end{aligned}$$

for a doubly centered matrix *B*

$$\begin{aligned} B = -\frac{1}{2} \left( I_N - \frac{1}{N} 1_N 1_N^\top \right) D^{(2)} \left( I_N - \frac{1}{N} 1_N 1_N^\top \right) , \end{aligned}$$

where $D^{(2)}$ is an element-wise square of matrix *D*. We note that the minimizer of strain function is analytically available via eigen-decomposition of the doubly centered matrix *B* and the embedding thereof is identical to that of PCA.

One limitation of classical MDS is that the method assumes the data dissimilarity *D* be given by standard $L_2$ norm in Euclidean space, which diminishes the interpretability of the attained embedding. Metric MDS provides an alternative approach by minimizing the stress function

$$\begin{aligned} Stress(X_{1:N}) = \sqrt{\sum _{i\ne j} (D_{ij} - \Vert X_i - X_j \Vert )^2}. \end{aligned}$$

The method aims at finding an optimal embedding that maximally preserves the pairwise distance structure regardless of the original metric space. As no closed-form solution exists for the stress minimization, we use the SMACOF algorithm^53^ that optimizes the cost objective by finding a majorizing function and updating an iterate by a minimizer of the majorizing function until convergence.

PGA is an adaptation of the principal component analysis (PCA)^54^ to the manifold setting. The core idea of PGA is to utilize the property of tangent space as a vector space. We briefly describe the procedure as follows. Once the Fréchet mean $\hat{C} \in {\mathscr {C}}_{++}^n$ is computed, every observation $C_1,\ldots , C_N$ is projected onto the tangent space $T_{\hat{C}} {\mathscr {C}}_{++}^n$ using the logarithm map $U_i = log_{\hat{C}} C_i$. The native version of PGA constructs an empirical covariance matrix $\Sigma = \sum _{i=1}^N U_i U_i^\top / N$ onto which eigen-decomposition is applied. If we denote eigen-pairs as $\lbrace \lambda _i, v_i \rbrace$ for $i=1,\ldots , \text {dim}({\mathscr {C}}_{++}^n)$, local coordinates for an observation *i* are given by $y_i = [v_1|\ldots |v_k]^\top log_{\hat{C}} (C_i) \in \mathbb {R}^k$. As the name suggests, PGA considers loadings to be projected basis onto the manifold via exponential map $\lbrace exp_{\hat{C}} (v_i)\rbrace$. Unlike conventional PCA on Euclidean space, the loadings, also known as principal geodesics, are not orthogonal in that they should be regarded as an approximate basis in the vicinity of the Fréchet mean.

## Results

### Simulation 1: Fréchet mean

In order to see whether the quotient geometry of the correlation manifold is a convincing alternative, we compared the effectiveness of Fréchet means under different geometries. We assume a simple multivariate Gaussian model $\mathcal {N}(0_5, I_5)$ in $\mathbb {R}^5$ as a generating process of the data and draw 50 observations for sample correlation computation to assure positive definiteness. At each iteration, 20 sample correlation matrices $C_1, \ldots , C_{20}$ are generated and different means are obtained, including (1) Fréchet mean on ${\mathscr {C}}_{++}^n$, (2) Fréchet mean on ${\mathscr {S}}_{++}^n$ with AIRM geometry and (3) its projection onto ${\mathscr {C}}_{++}^n$ by a submersion $\Pi$, and (4) Euclidean mean $\sum _{i=1}^{20} C_i / 20$. We recall that the third approach to combine SPD geometry and projection was extensively tested for functional connectivity analysis and showed good results on several inferential tasks^55^.

**Figure 1:**
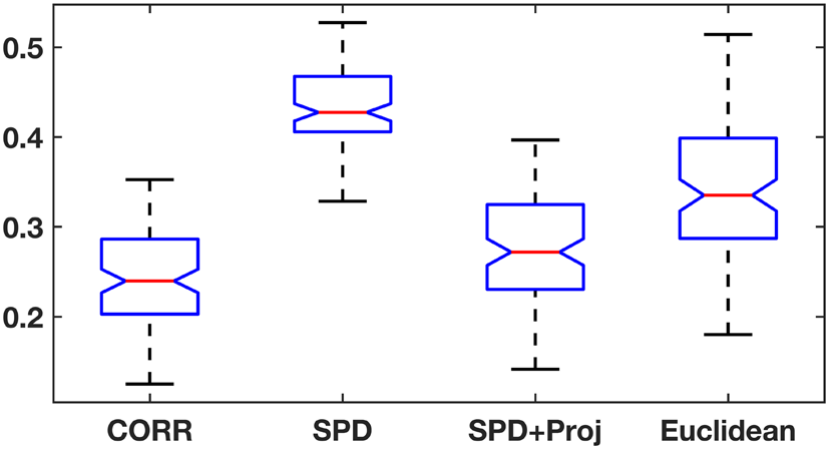
Comparison of error distributions for means of simulated correlation matrices under different geometries.

Figure 1 shows empirical error distribution of different means against the identity matrix measured in the Frobenius norm. The quotient geometry for the correlation matrix shows the least degree of error while AIRM performed poorly. This phenomenon is not surprising since the Fréchet mean of correlation matrices under AIRM is, in general, not even a correlation matrix. On the other hand, the post-hoc projection of SPD mean estimate onto ${\mathscr {C}}_{++}^n$ shows compatible results with that of the correlation manifold structure.

**Figure 2:**
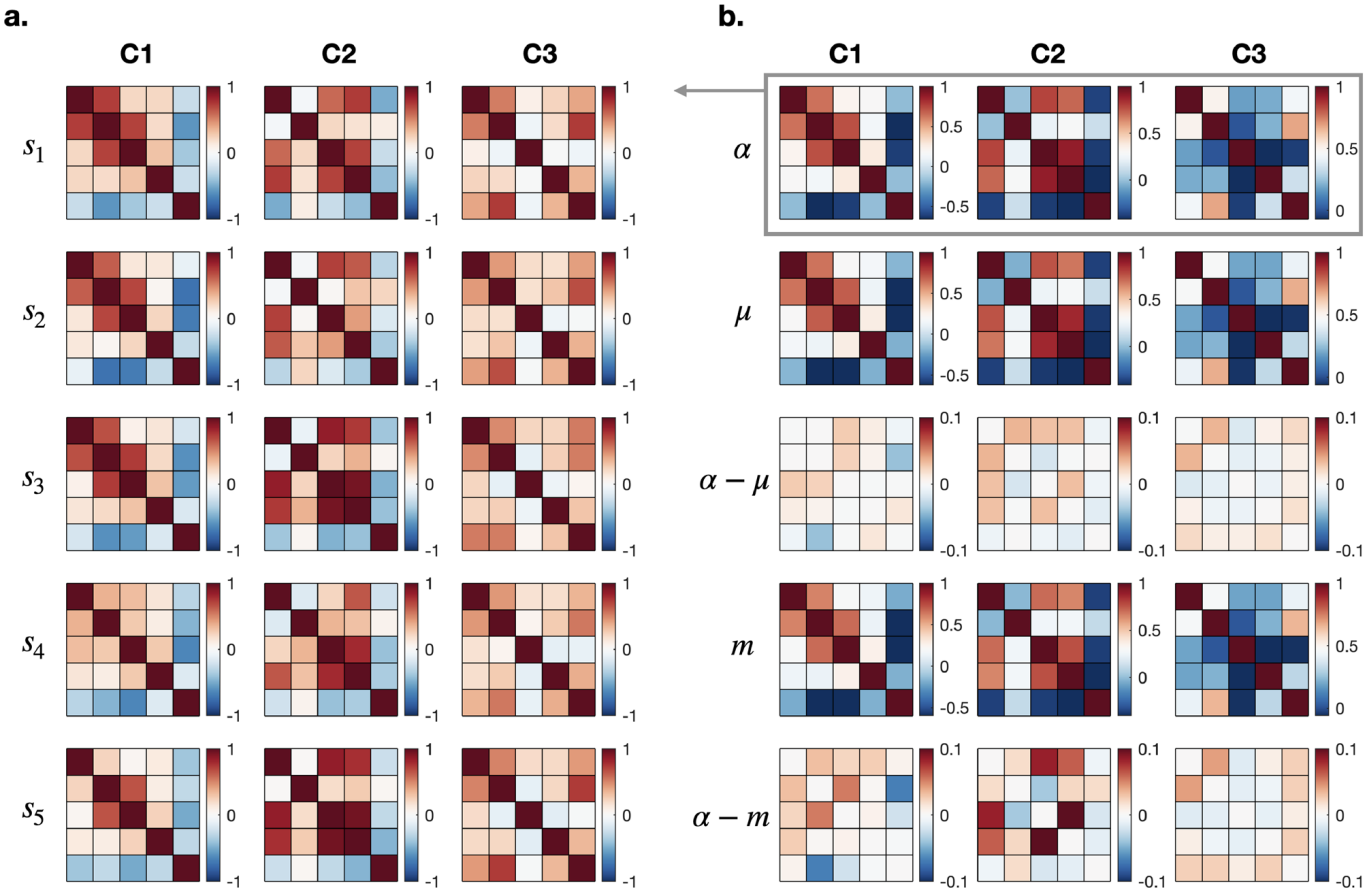
Comparison of the geometric mean ($\mu$) and Euclidean average (*m*) for 30 samples generated from the three model correlation matrices (C1, C2, and C3) shown in the first row of (**b**), enclosed by a gray rectangle. Among the thirty samples slightly deviated from each model correlation matrix obtained by adding noise (the model matrix is denoted as $\alpha$ for C1, C2, and C3), five exemplary samples for each model correlation matrix are presented in (**a**). The third and fifth rows in (**b**) indicate the element-wise differences from the model correlation matrix ($\alpha$). By visual analysis, greater differences are found in the Euclidean average compared to the geometric mean.

We then compared the Fréchet mean and the Euclidean average. We generated 30 samples from three model correlation matrices by adding noise with a standard deviation of 1 in the geometric tangent space. The model correlation matrices were derived from human connectivity matrices obtained from resting-state fMRI. These matrices are mutually distant from each other under all of Euclidean, SPD, and correlation geometries. Figure 2a shows five samples derived from the three model correlation matrices, which are presented in the first row of Fig. 2b. The second and fourth rows indicate the Fréchet mean and the Euclidean average (mean of each element of the correlation matrix). The third and fifth rows indicate the difference between the model correlation matrix and the Fréchet mean and the Euclidean average.

Next, we compared wall-clock time of Fréchet mean computation under correlation and SPD geometry of AIRM with varying number of observations and dimensionality. The results are summarized in Table 1, where correlation structure puts a more immense computational burden than AIRM. This result is due to the logarithmic map on the correlation manifold, where a minimization problem over ${\mathscr {D}}_{++}^n$ needs to be solved for every observation at each iteration. One remedy is to limit the number of iterations for an internal optimization problem at the cost of using a suboptimal diagonal matrix. We empirically witnessed that the approximate solution did not sacrifice the performance much while saving a considerable amount of time.

**Table 1:** Average computational time (in s) to compute Fréchet mean of *N* correlation matrices of size $(n\times n)$ under correlation and SPD geometry.

		$n=5$	$n=10$	$n=15$	$n=30$
$N=20$	CORR	0.183	0.487	0.839	2.249
	SPD	0.008	0.016	0.035	0.073
$N=50$	CORR	0.383	0.988	1.690	5.365
	SPD	0.017	0.032	0.069	0.171
$N=100$	CORR	0.813	2.132	3.387	10.743
	SPD	0.035	0.066	0.145	0.341

### Simulation 2: dimension reduction and cluster analysis

In this example, we generated 90 correlation matrices consisting of three populations of size 30 for each of the three model matrices used above (C1, C2, and C3) in ${\mathscr {C}}_{++}^5$.

**Figure 3:**
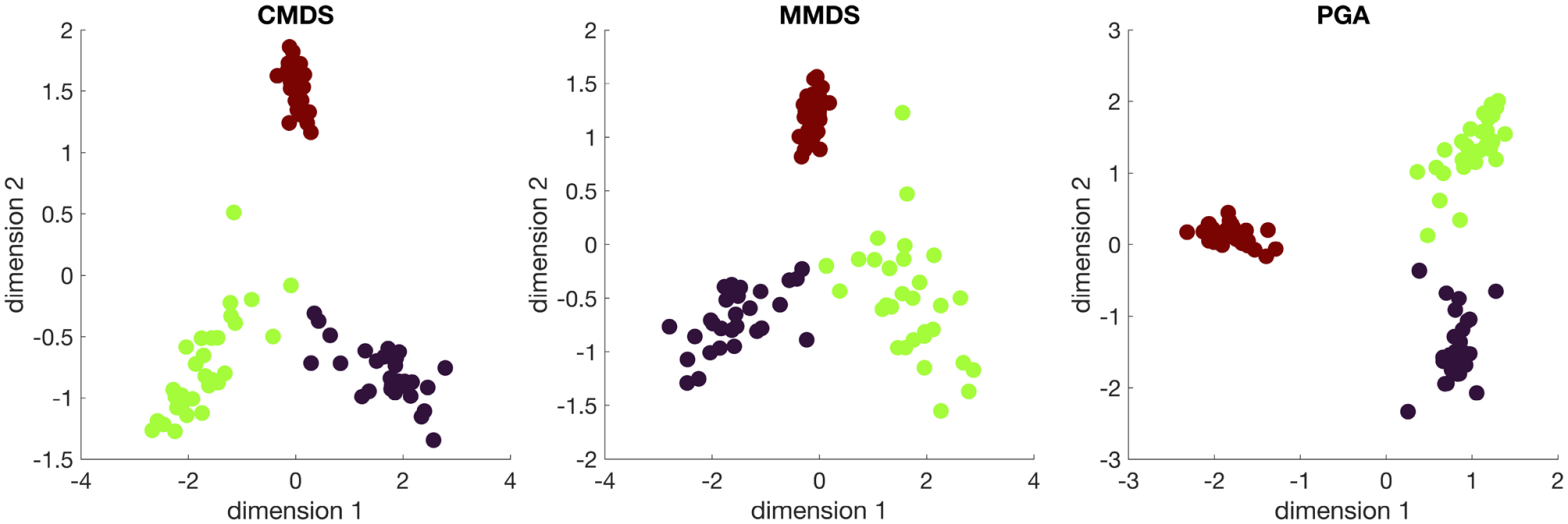
Embeddings of 90 correlation matrices from three model correlation matrices (C1, C2 and C3) by dimension reduction algorithms of classical multidimensional scaling (CMDS), metric multidimensional scaling (MMDS), and principal geodesic analysis (PGA).

First, we applied three dimension reduction algorithms, i.e., classical multidimensional scaling (CMDS), metric multidimensional scaling (MMDS), and principal geodesic analysis (PGA) to the generated data from the three model correlation matrices. Embeddings shown in Fig. 3 assert validity of the presented algorithms.

Next, we validate three clustering algorithms on the generated data with varying numbers of clusters *K*. Since the true number of clusters in this simulation is 3, a reliable clustering algorithm is expected to return a coherent partition when $K=3$ and discourage others. We summarized the results in Table 2 where the Silhouette score and CH index are used to quantify the effectiveness of clustering algorithms under the choice of *K*. It is not surprising that all methods showed the pattern that a peak appears for $K=3$ while discriminating $K=2$ and $K=4$ since the data is well separated, as shown previously.

**Table 2:** Empirical cluster quality measures for cluster partitions obtained by proposed algorithms under varying number of clusters *K*.

	Silhouette			CH index		
*K*	2	3	4	2	3	4
*k*-means	0.46	0.73	0.51	0.98	3.82	2.71
*k*-medoids	0.47	0.76	0.55	0.98	6.92	4.60
Spectral clustering	0.46	0.75	0.51	0.98	6.92	4.58

CH indices are scaled by 0.01 for demonstration purpose.

### Simulation 3: hypothesis testing

We tested the effectiveness of two nonparametric tests for equality of distributions. For model correlation matrices C1, C2, and C3, we generated 100 perturbed observations and applied the two tests in a pairwise manner on three samples. For each setting, we ran $10^5$ permutations to make the Monte Carlo procedure credible. In all pairwise comparisons, both tests showed extremely significant *p* values of $10^{-5}$. This means that every resampled statistic was smaller than that of the data $\hat{T}_{m,n}$. This implies that both tests can distinguish two well-separated empirical measures on the correlation manifold.

**Figure 4:**
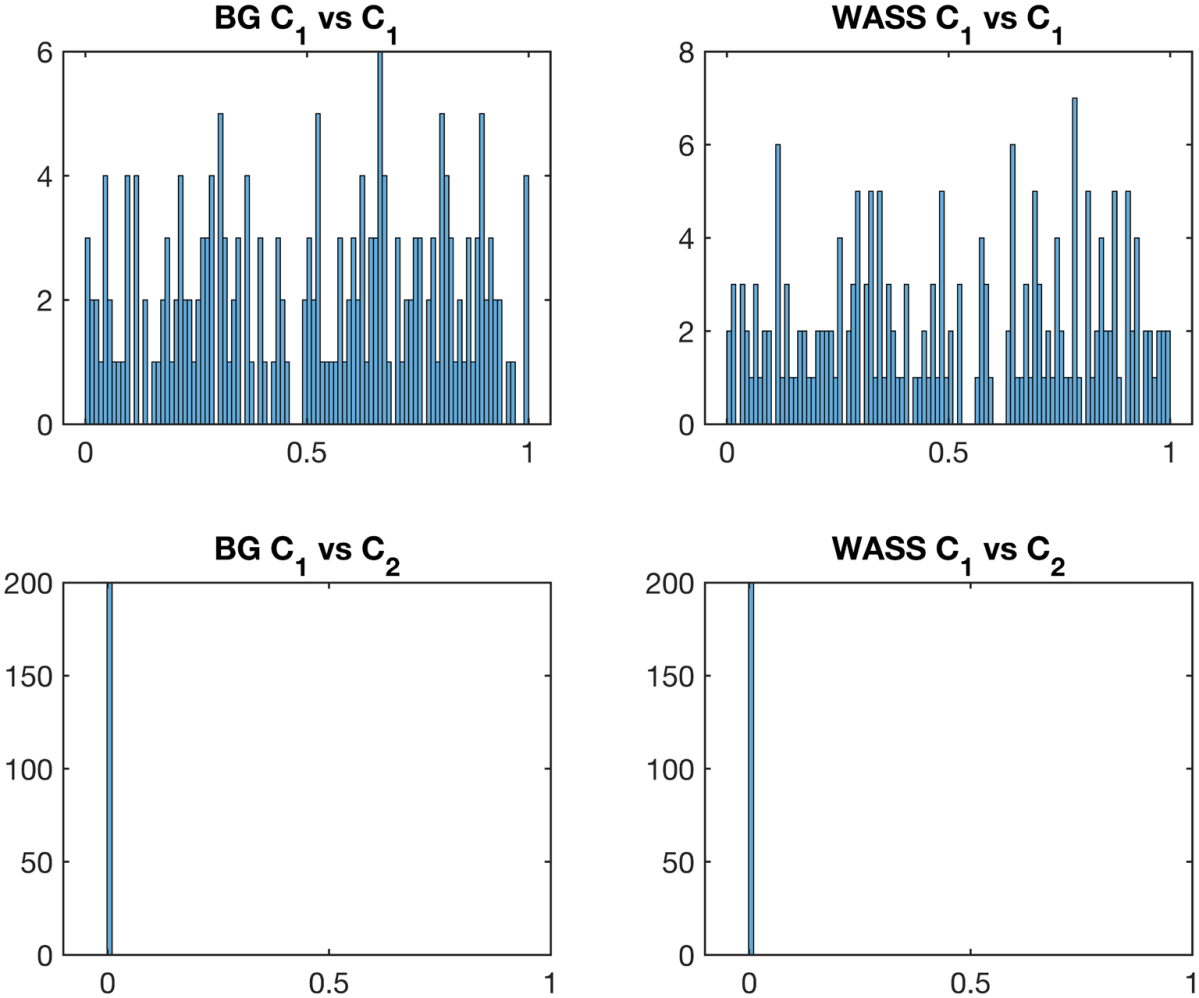
Hypothesis testing for two sets of correlation matrices driven from a model correlation matrix $C_1$ or from two different model correlation matrices $C_1$ and $C_2$. Each set contains 30 random samples from the model correlation matrix. Empirical distributions of 200 *p* values by Biswas–Ghosh (BG) and Wasserstein (WASS)-based tests are presented.

Next, we visualized an empirical distribution of *p* values under the null hypothesis of equal distributions, which is known to follow a uniform distribution on [0, 1]^56^. For a model correlation matrix $C_1$, two sample sets were drawn with noise, each consisting of 30 observations. Figure 4 shows the histogram of *p* values from 200 testing of two sets comprising random 30 samples (per set) derived from a group $C_1$ and the two different groups (i.e., $C_1$ and $C_2$), using Biswas–Ghosh (BG) and Wasserstein (WASS)-based tests.

### Real data analysis: EEG motor imagery dataset

In this example, we show efficacy of our proposed framework on the EEG motor movement/imagery dataset^57^, which is publicly available at PhysioNet^58^. To briefly describe, the dataset is a collection of EEG recordings of one and two minutes duration from 109 volunteers. Subjects performed 14 experimental runs of different motor and imagery tasks whose neural activities were recorded with 64-channel EEG using the BCI2000 system^59^. Among many tasks, we are interested in distinguishing the imagery operation of one or both fists from those of feet under the correlation-based functional network perspective.

To show validity of the proposed method, we took recordings from a randomly chosen single subject (S007) and extracted time series for tasks involving both fists and both feet. For preprocessing, we first selected 32 out of 64 channels (Fc5, Fc1, Fc2, Fc6, C3, Cz, C4, Cp5, Cp1, Cp2, Cp6, Fpz, Af7, Afz, Af8, F5, F1, F2, F6, Ft7, T7, T9, Tp7, P7, P3, Pz, P4, P8, Po3, Po4, O1, O2), removing the other 32 channels as they are marked as bad channels whose signals are either flat or show excessive signal-to-noise ratio^57^. We applied a Butterworth IIR band-pass filter with cutoffs at 7 and 35Hz. For network-based analysis, we epoched the filtered signals from every stimulus onset to a second after the onset (161 samples). Pearson correlation coefficients were constructed from the epoched signals. This led to a total of 45 correlation matrices where 23 are for feet, and 22 are for fists. Each correlation matrix is a $32\times 32$ matrix whose rows and columns correspond to 32 remaining channels. All 45 matrices were verified to be full-rank so that they are indeed proper objects for analysis on the correlation manifold.

**Figure 5:**
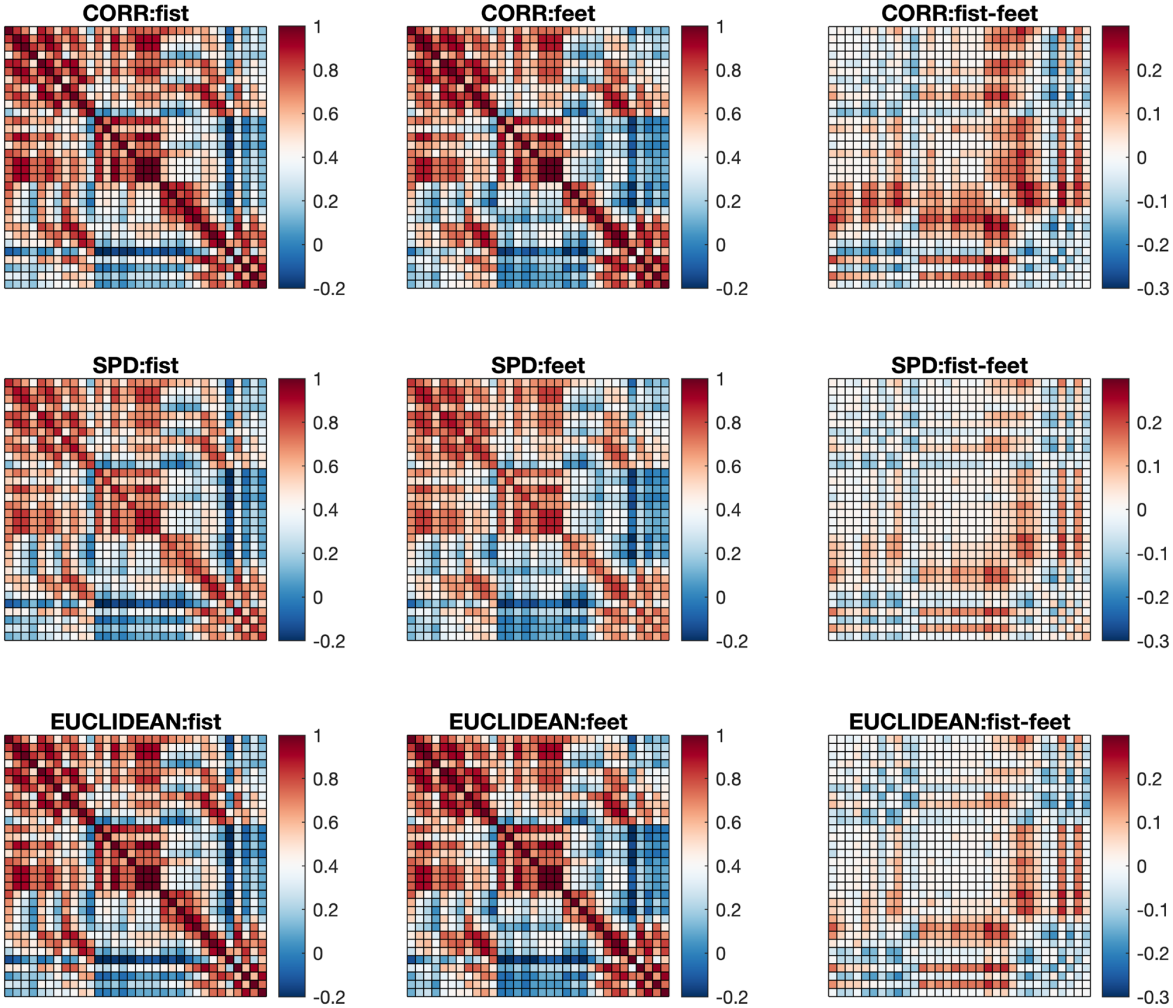
Fréchet mean of correlation matrices from fists and feet classes. Rows indicate a choice of geometric structure by viewing the matrices as elements of the correlation manifold (top), the SPD manifold (middle), and the Euclidean space (bottom). First two columns are Fréchet means for the fists and the feet classes, respectively. The last column is difference in Fréchet means of two classes.

We computed Fréchet means of two classes under different geometries as a preliminary step in exploratory data analysis. As shown in Fig. 5, it appears clear that all three geometries were somehow capable of capturing distinct patterns across different classes, which is an expected phenomenon considering the nature of the data. However, when we consider differences in two mean matrices, the correlation manifold identified local heterogeneity well compared to the other two geometries.

**Table 3:** Empirical *p* values on two classes of correlation matrices obtained from the Wasserstein (WASS) and Biswas–Ghosh (BG)-based permutation tests under three geometries.

Algorithms	Geometries		
	CORR	SPD	Euclidean
WASS	0.019*	0.056	0.108
BG	0.037*	0.095	0.144

Numeric values with an asterick * indicate the null hypothesis of equal distributions is rejected at the significance level of $5\%$.

We also performed hypothesis testing of equal distributions on the two-class correlation data under different geometries. For both Biswas–Ghosh (BG) and Wasserstein (WASS)-based tests, we ran $10^5$ permutations to guarantee the credibility of the resampling-based procedures. As summarized in Table 3, both tests showed significant empirical *p* values upon the correlation manifold structure at the significance level $\alpha =5\%$, suggesting that two empirical measures are statistically distinguishable on the correlation manifold only. An interesting observation is that although the other two geometries did not indicate a significant difference between the two classes, the SPD geometry showed dominant results against the Euclidean space assumption, and its empirical *p* value by the Wasserstein-based test is close to the cutoff value. This result aligns with our previous observation in the sense that a Riemannian approach can be a proper alternative that reflects intrinsic nonlinearity on the space of functional connectivities^55^. The statement, however, does not necessarily preclude absolute dominance of a specific geometry against the others.

**Figure 6:**
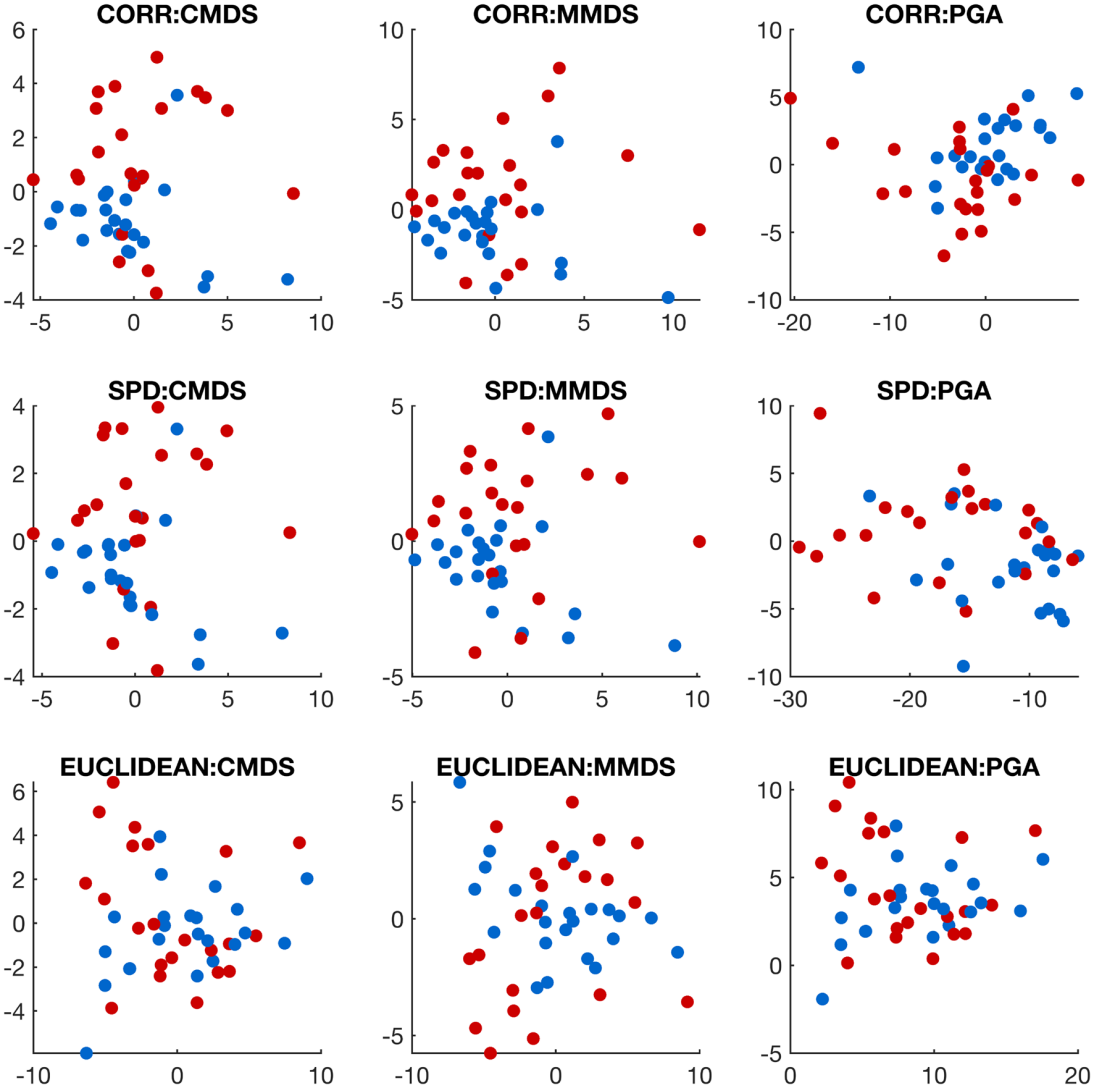
Embeddings onto 2-dimensional Euclidean space for a collection of motor imagery correlation matrices. Rows correspond to a choice of geometric structure by viewing the matrices as elements of the correlation manifold (top), the SPD manifold (middle), and the Euclidean space (bottom). Columns represent algorithms used under each geometry, including classical multidimensional scaling (left), metric multidimensional scaling (center), and principal geodesic analysis (right). Colors represent two classes of fists (in red) and feet (in blue).

Lastly, we demonstrated how a choice of manifold structure affects its low-dimensional embedding. In Fig. 6, we presented 2-dimensional embeddings from the algorithms we introduced in our paper under three different geometries. At a glance, all embeddings seem to show entangled patterns for two separate classes. However, the correlation manifold shows the largest degree of distinction between two classes across all algorithms, while the Euclidean assumption does little separation. Similar to the previous experiments with other tasks, it is worth mentioning that the SPD geometry locates between the other two geometries. This partially supports that the SPD geometry may still be employed on its submanifold of correlation matrices at the cost of performance and concerns in choosing a more suitable geometry.

## Discussion

As a basic tool for functional network analysis, the correlation matrix contains more information as a whole than the sum of independent pairwise correlation coefficients. Thus, the correlation matrix may well be dealt with as manifold-valued objects with corresponding geometric structures. In recognition of the importance for operation over the proper manifold, a growing number of studies have analyzed the correlation matrix under the SPD geometry^19–23^.

One critical limitation to considering correlation matrices simply as objects on SPD manifold is that operations with correlation matrices do not necessarily take the form of the correlation matrix and thus demand a post-hoc step to constrain unit diagonal elements. In our previous study^55^, we iteratively normalized output matrices (derived from the correlation-matrix operation) into correlation matrices at each intermediate step. Although this heuristic works well in most cases as shown in our simulation study, this approach is not an exact solution. The current study resolves this naive solution by implementing operations over the correlation matrix manifold space known as elliptope^24^, a mathematical space whose computational routines have been little known^25,26^. Two recent seminal works^27,29^ make the construction of the correlation matrix space computationally feasible and lay the basis of the current paper.

Based on these recent developments in the quotient geometry of the correlation manifold, we numerically implemented computational operations over the correlation matrix space. We then presented the most fundamental analyses and inferential algorithms for practical use in functional network analysis, including measures of central tendency, cluster analysis, hypothesis testing, and low-dimensional embedding. The simulation result suggests the effectiveness of analysis on the correlation manifold. In our simulation, the SPD-based approach with post-hoc normalization shows comparable performance against the correlation-based approach. Nevertheless, the proposed framework is expected to be theoretically more sound. For example, our experiment, as shown in Fig. 2, revealed that even the most straightforward task of finding the mean over perturbed correlation matrices around the identity matrix benefits from the dedicated geometric structure. Our real data example demonstrated significance of our proposed approach in localizing differentiating inter-region connections between two classes. Furthermore, hypothesis testing and low-dimension embedding examples even provided grounds to argue that the correlation manifold was the only geometry that revealed difference in two clearly distinct classes of correlation matrices. It should be noted that the advantage of functional network analysis under the correlation-manifold framework may not always be conspicuous compared to the SPD manifold or Euclidean treatment in machine learning or statistical analysis. However, it is mathematically more exact and consistent than the SPD framework in considering inter-dependence among edges in the functional brain network.

Despite its mathematical consistency and superb performance, there remains a number of issues to be addressed which set major directions for future studies. First, the correlation manifold structure provides an effective geometric framework at the cost of increased computational burden compared to that of the ambient SPD manifold. In our experiments, we observed that pertained computations were within a reasonable time range for a general network size of 30 (Table 1). Still, its nested iterative nature has its potential to hamper wider use in practice. This necessitates to devise a set of numerical routines that can dramatically reduce computational costs, which we view as an invaluable opportunity. The current correlation analysis methods also inherit the same practical limitation of the SPD in the neuroimaging analysis. When the number of scans for resting-state fMRI is small compared to the network size, correlation matrices are likely to be rank-deficient. In this case, one may consider estimating correlation matrices under assumptions like sparsity, strict non-singularity, and others^60,61^.

Besides the functional network analysis, the correlation manifold would be applied to other neuroimaging research fields such as Representational Similarity Analysis (RSA). RSA is a multivariate technique that explores brain processes according to similarity matrices of the brain responses to the different types of stimuli^62,63^. In the RSA, the similarity is generally defined in terms of the correlation matrix. Shahbazi et al.^64^ showed more accurate quantification of representational similarities over the SPD manifold than the Euclidean operation. Considering the similarity definition based on the correlation matrix, the correlation manifold proposed in the current study would be a more appropriate choice for RSA.

The application of the correlation manifold is not restricted to brain research. It extends to any other research areas that utilize correlation matrices. For example, correlation matrix is a popular form of data in financial markets^65–67^. The geometric approach to correlation-based functional network analysis is still nascent in the neuroimaging and other related research communities. We wrapped all the algorithms in the current paper as a toolbox of MATLAB (Mathworks, Inc. USA) called CORRbox, which is freely available on GitHub (https://github.com/kisungyou/papers). We expect that CORRbox would be of great use in analyzing matrix representations of functional networks by taking advantage of exact representations and operations over the proper manifold of the correlation matrices.

## Supplementary Information

Supplementary Information.

## Data Availability

The CORRbox, a MATLAB toolbox for learning with data on the correlation manifold, is publicly available at the github repository (https://github.com/kisungyou/papers) along with examples.
