# Supplementary material for: Geometric learning of functional brain network on the correlation manifold
Source: Sci Rep. 2022 Oct 22;12:17752. doi: 10.1038/s41598-022-21376-0 (PMC9588057; doi:10.1038/s41598-022-21376-0)
Supplement: Supplementary file 1 — Supplementary Information. [file 41598_2022_21376_MOESM1_ESM.pdf]

Geometric learning of functional brain network on the correlation manifold

Kisung You[1,2] and Hae-Jeong Park[2,3,4,*]

[1]Department of Internal Medicine, Yale School of Medicine, New Haven, CT, USA
[2]Center for Systems and Translational Brain Science, Institute of Human Complexity and Systems Science, Yonsei University, Seoul, Republic of Korea
[3]Graduate School of Medical Science, Brain Korea 21 Project, Department of Nuclear Medicine, Psychiatry, Yonsei University College of Medicine, Seoul, Republic of Korea
[4]Department of Cognitive Science, Yonsei University, Seoul, Republic of Korea
[*]Corresponding author : parkhj@yonsei.ac.kr

1 Elements of Riemannian geometry

A manifold $\mathcal{M}$ is a topological space such that the vicinity of a point on $\mathcal{M}$ resembles Euclidean space. A Riemannian manifold $(\mathcal{M}, g)$ is a smooth manifold with a smoothly-varying positive-definite inner product g_p on the tangent space $T_p\mathcal{M}$.

A critical characteristic of the Riemannian manifold is that the structure is endowed with familiar concepts such as angle or distance that satisfy metric properties. However, it is not a vector space, meaning that even elementary operations like addition and multiplication by a scalar are not usually available. Therefore, it is crucial to replace conventional operations in Euclidean space with those that abide by the machinery of Riemannian geometry. Rooted from such motivation, we introduce some concepts and definitions, focusing on intuitive explanation without rigorous mathematical details.

A tangent space $T_p\mathcal{M}$ is a set of tangent vectors that are derivatives of curves crossing a point $p \in \mathcal{M}$. When we say a manifold locally resembles Euclidean space, it generally refers to a tangent space as a vector space. At every point $p \in \mathcal{M}$, we have the inner product g_p, called Riemannian metric, that locally endows an inner product structure on the tangent space. Formally speaking, the positive definiteness is that for any non-zero vector $v \in T_p\mathcal{M}$, $g_p(v, v) > 0$. Also, it varies smoothly in the sense that a map $p \mapsto g_p(u, v)$ for any $u, v \in T_p\mathcal{M}$ is a smooth function. Exponential and logarithm maps are two important operations that connect a tangent space and a manifold. Exponential map $exp_p(\cdot) : T_p\mathcal{M} \rightarrow \mathcal{M}$ gives a unique shortest curve called geodesic from p in the designated direction. If we take a tangent vector $v \in T_p\mathcal{M}$, exponential map defines a ray emanating from p in the direction of v so that the operation results in a point on $\mathcal{M}$. An inverse of exponential map is called logarithm map; for two points $p, q \in \mathcal{M}$, $\log_p(q) \in T_p\mathcal{M}$ returns a tangent vector that can be pulled back to q by an exponential map. Lastly, geodesic distance for two points on $\mathcal{M}$ is measured by length of the shortest curve connecting the two.

2 Geometry of SPD manifold

Popular representations of functional connectivity such covariance, correlation, and precision matrices all belong to the space of SPD matrices which we denote as $\mathcal{S}^n_{++}$.

Definition 1. *$\mathcal{S}^n_{++}$ is a space of $(n \times n)$ symmetric and positive-definite matrices*

$$\mathcal{S}^n_{++} = \{X \in \mathbb{R}^{n \times n} \mid X = X^\top,\ rank(X) = n\},$$

where $rank(A)$ is the matrix rank and dimension of $\mathcal{S}^n_{++}$ is $n(n+1)/2$.

The space of SPD matrices beyond Euclidean regime has long been interest due to its popularity (Bhatia; 2009). Focusing on different aspects of the space, many proposals have been made to formulate non-Euclidean geometry of SPD space where the distance between two SPD matrices is defined by Cholesky decomposition (Dryden et al.; 2009), log-Euclidean Riemannian metric (Arsigny et al.; 2007), Bures-Wasserstein geometry (Takatsu; 2011), or Jensen-Bregman LogDet divergence (Cherian et al.; 2013) to name a few.

Among many geometric structures, the affine-invariant Riemannian metric (AIRM) is one of the most well-known machineries on $\mathcal{S}^n_{++}$ because it fully characterizes the space of SPD matrices as a Riemannian manifold hence the shortest-path curve between two matrices is naturally expressed by the geodesic (Pennec;

2006). For a point $P \in \mathcal{S}^n_{++}$ and tangent vectors $U, V \in T_P\mathcal{S}^n_{++}$, AIRM defines a Riemannian metric by

$$g_P(U,V) = \mathrm{Tr}(P^{-1}UP^{-1}V),$$

where P^{-1} is a standard matrix inverse and Tr is a trace operator. AIRM is also endowed with closed-form formula for exponential and logarithm maps. For two points $P, Q \in \mathcal{S}^n_{++}$ and a tangent vector $V \in T_P\mathcal{S}^n_{++}$, exponential map $exp_P : T_P\mathcal{S}^n_{++} \rightarrow \mathcal{S}^n_{++}$ is given by

$$exp_P(V) = P^{1/2} Exp(P^{-1/2}VP^{-1/2})P^{1/2},$$

and its inverse logarithm map, is obtained by

$$log_P(Q) = P^{1/2} Log(P^{-1/2}QP^{-1/2})P^{1/2},$$

where $Exp(\cdot)$ and $Log(\cdot)$ are matrix exponential and logarithm, respectively (Hall; 2015). Finally, the geodesic distance $d : \mathcal{S}^n_{++} \times \mathcal{S}^n_{++} \rightarrow \mathbb{R}_+$ is computed as

$$d^2(P,Q) = g_P(log_P Q, log_P Q) = \|Log(P^{-1}Q)\|_F^2,$$

where $\|A\|_F = \sqrt{\mathrm{Tr}(A^\top A)}$ is a Frobenius norm. This formula arises from close relationship between the geodesic distance and the Riemannian metric (Pennec; 2006; Pennec et al.; 2020).

3 Distance Computation on $\mathcal{C}^n_{++}$

For two correlation matrices $P, Q \in \mathcal{C}^n_{++}$, the geodesic distance is defined as

$$d^2_{\mathcal{C}^n_{++}}(P,Q) = \min_D d^2_{\mathcal{S}^n_{++}}(P, DQD) \quad \text{for } D \in \mathcal{D}^n_{++},$$

where $\mathcal{D}^n_{++}$ is a collection of $(n \times n)$ diagonal matrices with positive entries and $d_{\mathcal{S}^n_{++}}$ is the geodesic distance in the SPD manifold under AIRM. David (2019) proposed a Riemannian gradient descent algorithm over $\mathcal{D}^n_{++}$ by the followings, which we introduce for completeness.

1. Initiate $D^{(0)} = I_n$ and set a stepsize $\tau > 0$.
2. Iterate until convegence

$$\Delta^{(t)} = I_n \bullet \mathrm{Symm}[D^{(t)} Log(QD^{(t)}P^{-1}D^{(t)})],$$
$$D^{(t+1)} = D^{(t)} Exp(-2\tau(D^{(t)} \backslash \Delta^{(t)})),$$

where $A \bullet B$ is a hadamard product, i.e., $[A \bullet B]_{ij} = A_{ij}B_{ij}$, an operator $\mathrm{Symm}[A] = (A + A^\top)/2$ extracts symmetric component of a given matrix, and backslash notation for $A \backslash B = A^{-1}B$. The presented algorithm iterates over a path over $\mathcal{D}^n_{++}$. First, the gradient $\Delta^{(t)}$ is a hadamard product of an identity and some matrix so that all off-diagonal entries are zeros. Since $D^{(t)}$ and $\Delta^{(t)}$ are both diagonal, the matrix exponential term is also diagonal, and so is the next iterate $D^{(t+1)}$. One may employ a line search to control the step size τ in an adaptive manner. However, a class of line search methods requires evaluation of the cost function repeatedly for a single update, which can add extra computational costs at exponential scale as the dimension gets larger due to matrix exponential and logarithms. Therefore, we stick to the constant step size rule as originally proposed (David; 2019), with a small value of $\tau \in (0, 1]$. In our experiments, we observed that any values in the range showed almost equivalent performance in geodesic distance computation for a reasonably concentrated empirical measure.

References

Arsigny, V., Fillard, P., Pennec, X. and Ayache, N. (2007). Geometric Means in a Novel Vector Space Structure on Symmetric Positive-Definite Matrices, *SIAM Journal on Matrix Analysis and Applications* **29**(1): 328–347.

Bhatia, R. (2009). *Positive Definite Matrices:*, Princeton University Press.

Cherian, A., Sra, S., Banerjee, A. and Papanikolopoulos, N. (2013). Jensen-Bregman LogDet Divergence with Application to Efficient Similarity Search for Covariance Matrices, *IEEE Transactions on Pattern Analysis and Machine Intelligence* **35**(9): 2161–2174.

David, P. (2019). *A Riemannian Quotient Structure for Correlation Matrices with Applications to Data Science*, PhD Thesis, Claremont Graduate University.

Dryden, I. L., Koloydenko, A. and Zhou, D. (2009). Non-Euclidean statistics for covariance matrices, with applications to diffusion tensor imaging, *The Annals of Applied Statistics* **3**(3).

Hall, B. C. (2015). *Lie Groups, Lie Algebras, and Representations: An Elementary Introduction*, number 222 in *Graduate Texts in Mathematics*, second edition edn, Springer, Cham ; New York.

Pennec, X. (2006). Intrinsic Statistics on Riemannian Manifolds: Basic Tools for Geometric Measurements, *Journal of Mathematical Imaging and Vision* **25**(1): 127–154.

Pennec, X., Sommer, S. and Fletcher, T. (2020). *Riemannian Geometric Statistics in Medical Image Analysis*, Academic Press, San Diego.

Takatsu, A. (2011). Wasserstein geometry of Gaussian measures, *Osaka Journal of Mathematics* **48**(4): 1005–1026.
